# Symposium—“The Necessity of an Early Exploratory Incision in Obscure Abdominal Conditions”

**Published:** 1913-12

**Authors:** 


### SYMPOSIUM—"THE NECESSITY OF AN EARLY EXPLORATORY INCISION IN OBSCURE ABDOMINAL CONDITIONS."

Symptoms referrable to the stomach, pylorus and duodenum by Dr. W. F. Westmoreland, of Atlanta, who referred to the view of internists that cancer of the stomach was rarer than was believed by the surgeon because symptoms were rarely diagnostic in the early stages of the disease.

Laboratory methods are insufficient for diagnosis, and where cancer is looked for and exploratory incisions made the diagnosis is made of value. Careful investigations should be made where the exploratory incision was adopted, for symptoms warranting same are usually vague. To wait for clinical diagnosis will render surgical treatment a palliative measure. If cancer of stomach is not found after exploratory incision, some remedial surgical condition can be found.

Symptoms referrable to the gall, bladder and liver were discussed by Dr. G. H. Noble, of Atlanta, who said in substance that exploratory operations were very common twenty-five years ago, but not so now because of the various expert methods of diagnosis. Prominent surgeons are not satisfied with present methods of diagnosis of diseases of the stomach, and are returning to the method of exploratory incisions. We should not, however, discredit present methods of diagnosis, which may have a corroborative, if not positive, value.

Yet it is an unwise precedent to make when we say that an exploratory incision must be resorted to without reservation. The exploratory incision should be adopted for diagnosis after faithful study and careful analysis.

The cold or amebic abscesses of the liver can only be diagnosed by early incision.

An incision is not necessarily exploratory provided the surgeon has a general idea of pathology before operation.

Symptoms referrable to the appendix and cecum were discussed by Dr. F. W. McRae, of Atlanta.

It is rarely necessary to make an exploratory incision for troubles about the appendix and cecum, and the case of a physician was cited who had had various diagnoses made in this country and abroad, and an exploratory incision revealed the presence of a fibroid appendix. Radiography and ureteral catheterization go a great way toward eliminating the necessity of exploratory incision. The practice of this procedure should be referred to expert laporatomists for obscure conditions and not the novice. Physicians in general are able to work an operable diagnosis of appendicitis.

Lanes Kink and Jackson's membrane justify exploratory incision.

Dr. W. W. Battey, Augusta, Ga.—

An exploratory operation is absolutely essential in a number of cases. Unfortunately it is difficult to induce a patient to allow such a procedure. The laity cannot appreciate the difficulties attendant upon a correct diagnosis and unless some definite condition presents itself, and they are so advised, exploratory operation appeals to them as a defeat on our part, and they seek other means of bringing about a cure.

Gall stones occur in individuals who never have colic. Associated with cholelithiasis we have obstructive conditions of bile ducts and cholecystitis. The symptoms of gall stone colic are pain referred to stomach, tenderness over gall bladder, and, as a rule, vomiting accompanies attack. Jaundice is not necessarily present and this occurs when there is an obstruction of common duct. Not all cases of gall stones demand exploratory incision. The necessity for operation should be judged from the type and degree of inflammatory disturbance caused by calculi either periodically or persistently.

Exploratory incision is justifiable in all cases of empyema of gall bladder if suspected by leucocytosis, fever rigors, etc. The gall bladder is often the seat of malignancy.

Tumors of liver and primary or secondary syphilis may give rise to tumors of large size. These tumors are either benign or malignant. Of the benign variety we have cysts, fibromata, adenamata, angiomata and syphilomata. Of malignant variety carcenoma or sarcoma.

Dysentery is the most common primary cause of liver abscess. It may also follow inflammatory affections in any part of portal system; it may result from direct injury to liver, wound infections and pyemia.

Exploratory punctures must never be done for abscess of liver unless we are prepared to do immediate operation.

Doctor James N. Ellis, of Atlanta, said:—Symptoms relative to the kidney, ureter and bladder, as a sub-title to the main topic of discussion at this meeting, "The Necessity of an Early

Exploratory Incision in Obscure Abdominal Conditions," is interpreted by me to refer to those affections of the kidney, ureter and bladder simulating abdominal conditions for which exploratory laparotomy might be indicated or considered desirable.

The elaborate diagnostic methods at our command, when systematically carried out, leaves but little to be desired in the way of measures that will assure us of a correct diagnosis in lesions of the genito-urinary organs. Palpation, percussion, complete laboratory examination of urine, cystoscopy, ureteral catheterization, segregation and radiography, when systematically and intelligently employed, will leave few, if any, affections of these organs sufficiently obscure to necessitate an exploratory incision.

An exploratory operation on these organs is almost a confession of being too indolent or too ignorant to practice the somtimes laborious and tedious details necessary to arrive at a correct pre-operative diagnosis. Hasty diagnosis here, as elsewhere, will lead to unnecessary operation by those afflicted with an excess of misdirected surgical energy. Fortunately, however, the custom is rapidly growing, in the more progressive surgical centers, of having, before operation is decided upon, numerous consultations between the surgeon, the internist and the specialist, and their conclusions are further and finally checked or confirmed by the results of exhaustive laboratory investigation. In the more provincial districts, where such facilities are not available and when urgent intervention seems necessary, operation "on suspicion" may occasionally be justifiable. The evolution and perfection of surgical technique, happily, robs the exploration of much of its erstwhile gravity; but a habit of practicing such procedures without great deliberation, ultimately brings the over-zealous surgeon and his profession into deserved disrepute.

There are, however, many lesions of the kidney, ureter and bladder presenting symptoms so closely simulating acute abdominal conditions for which exploration is occasionally necessary that, if we are not on guard, "on the lookout" for them, we may make the mistake of seeking to solve them surgically by an abdominal incision and exploration of the contents of the peritoneal cavity. But recently I had a case referred to me for an appendectomy which, upon investigation, was found to be a unilateral, right-sided acute nephritis. If we fail to carefully consider the genito-urinary system, an intermittent, unilateral hydronephrosis might easily be confused with appendicitis, cholecystitis or other intra-peritoneal lesions. Differentiation between nephro-pelvic lithiasis and cholelithiasis and between ureteral calculus and appendicitis is not infrequently necessary. Bacterial invasion of the upper urinary tract, pyelitis and haematogenous infections of the kidney and its pelvis may also need to be differentiated from acute abdominal lesions. Hydronephrosis has been, and may easily be, mistaken for a pseudo or true panceratic cyst, by an over-confident or careless observer. A distended urinary bladder has been mistakenly diagnosed as a broad ligament—or ovarian cyst!

It is enough to remember, however, that such conditions as these may closely simulate each other, to put us on our guard. Differentiation is easy with the means of diagnosis at our command and, upon investigation, the apparent confusion disappears.

There is one class of cases, however, in which immediate operation, of a semi-exploratory nature, may be imperative. I refer to suspected severe traumatism of kidney, ureter or bladder. In all traumatic injuries of the trunk, such as crushes, blows, and in puncture or penetrating wounds which might damage these organs, the patient should immediately be catheterized and, if blood appears in the urine, the source of the hemorrhage should at once be ascertained. If a small amount of bloody urine is obtained, suspect rupture of the bladder; if a large amount, suspect the kidney, and make an exploratory incision, if necessary, to determine the condition of the suspected organ. These cases,—rupture of kidney or bladder,—usually have early primary vomiting, once or twice, rarely more frequent. The patient with grave injuries of this kind, even severe enough to necessitate immediate surgical intervention if we are to save life, frequently does not immediately show the profound symptoms that one would expect, and to await their development would invite irremediable disaster.

In this connection, I would like also to refer to the symptoms of an acute, mechanical, postoperative hydronephrosis incident to the accidental ligation of the ureter during a pelvic or abdominal operation, and of the urgent necessity for its immediate relief. Essential diseases of the kidney, ureter and bladder, incapable of being correctly interpreted by patient investigation along the lines I have indicated and necessitating an exploratory incision for their elucidation, are so rare and infrequent that they may almost be classed as surgical curiosities; and I will leave the consideration of this phase of the subject to the specialists who will participate in this discussion.

---

Dr. Edgar G. Ballenger, of Atlanta, in discussing the "Necessity of Early Exploratory Incision in Obscure Abdominal Conditions," with special reference to the Genito-Urinary organs, expressed the opinion that when such exploratory incisions were sometimes necessary, fortunately modern diagnostic methods enabled one nearly always to make a correct diagnosis before operating.

With cystoscopy, ureteral catheterization, stylet, pyelography, X-ray examinations, etc., we can, as a rule, obtain accurate information as to the existing condition unless it be unusually rare. We are not, therefore, justified in all exploratory incision until after such exhaustive examinations have been made.

There are obscure conditions which cannot be diagnosed by the most careful tests. These, of course, require an exploratory incision. Several years ago I treated a patient with a hematuria from the rght kidney of sixteen years duration. Every test was negative. An exploratory incision also seemed negative, the right kidney being apparently normal, even when incised. As the previous ureteral catheterization had shown the left kidney to be of good functional capacity and that the blood came from the right kidney, I removed the right kidney.

The report fro mthe pathologist proved the condition to be probably a varicose condition of some of the veins on the papillae, which had produced the so-called essential hematuria. In this instance nothing but exploration could clear the situation and even then we were left in doubt. The previous examinations, however, indicated what should be done. The nephrectomy stopped the hemorrhage and the patient made an uneventful recovery. Dr. Ellis has covered the ground so well that I will not take any more of your time by discussing other rare affections that call for exploratory incisions.

Dr. J. Campbell gave a surgical clinic at the Atlanta Medical College at 8:30 A. M., Wednesday.

Dr. M. L. Boyd, Atlanta, said: The principal things to be said about exploratory operations on the kidney, bladder and ureter are concerning the various methods now employed which give us definite knowledge concerning the existing pathological lesions, that the necessity of making an exploratory incision rarely occurs, when they are used.

There are few conditions, however, which do require an exploratory operation to decide upon thier exact nature. Dr. Ballenger has already mentioned the rare cases of tumors of the pelvis of the kidney in which the only symptom was hematuria. Papilloma of the ureter also comes under this class, but we sometimes get with them symptoms of urinary obstruction and dilatation of the kidney pelvis as well as the bleeding.

The symptoms of hypernephroma of the kidney are at times also obscure since they may be simply an enlargement of the kidney with or without haematuria. The pre-operative correct diagnosis can often be made, but not always.

Further, there are certain congenital conditions of the kidney, such as cystic formations, which cannot be satisfactorily diagnosed previous to operation. A rare and interesting case of this kind was seen in a patient of Dr. Floyd McRae's where the cyst had largely replaced the kidney substance and extended well up under the liver.

Tuberculosis of the upper urinar ytract may so involve the bladder that it is impossible to exqmine the kidneys by the usual cystoscopic methods; some such cases might require an exploratory operation if it was suspected that only one kidney was involved or was destroyed.

There are practically no pathological conditions in the bladder requiring exploratory operation, except a few rare ones hardly worth while mentioning here.

Dr. Floyd W. McRae gave a surgical clinic at the iPedmont Sanatorium, at 8:30 a. m. Thursday.

Dr. S. T. Barnett held a gynecological clinic at Grady Hospital Thursday at 8:30 a. m.

Dr. F. K. Boland gave a clinic at Grady Hospital Wednesday at 10:30 a. m.

